# A case of encapsulated papillary carcinoma of the breast treated with emergency surgery due to sudden hemorrhage

**DOI:** 10.1016/j.ijscr.2019.10.048

**Published:** 2019-11-01

**Authors:** Kimiyasu Yoneyama, Motohito Nakagawa, Asuka Hara

**Affiliations:** Department of Breast Surgery, Hiratsuka City Hospital, Hiratsuka, Japan

**Keywords:** CT, computed tomography, EPC, encapsulated papillary carcinoma, MRI, magnetic resonance imaging, Emergency surgery, Breast cancer, Encapsulated papillary carcinoma of the breast, Haemorrhage, Oestrogen receptor, Progesterone receptor

## Abstract

**Introduction:**

Emergency surgery is rare in management of breast cancer. We report a case of encapsulated papillary carcinoma (EPC) of the breast where emergency surgery was performed because of unsuccessful control of hemorrhage.

**Presentation of case:**

A 62-year-old woman visited our hospital complaining of sudden bleeding from the right breast. She had been aware of the tumor for a year, but had left it unattended. It had been increasing in size rapidly for a few days before presentation. Computed tomography showed a hypervascular cystic tumor 9 cm in diameter. Bleeding was observed from a skin breach in the right breast, from which a clot was removed and the wound compressed with gauze. We attempted unsuccessfully to control the hemorrhage over the next 3 days, but the patient became anemic, so emergency surgery was performed to control the bleeding. Histopathology revealed the tumor as estrogen receptor- and progesterone receptor-positive EPC, with a human epidermal growth factor receptor 2 score of 0.

**Discussion:**

There have been only 5 reports of breast cancer treated with emergency surgery. EPC was previously considered an *in situ* lesion, but the lack of a myoepithelial layer at the lesion's periphery may represent a low grade or indolent form of invasive carcinoma and prognosis is usually good. However, if bleeding into the cyst occurs and hemostasis is difficult, emergency surgery should be considered.

**Conclusion:**

Emergency surgery is rare in breast cancer but should always be considered as an option when hemorrhage is not readily controlled.

## 1 Introduction

Emergency surgery is rare in the management of breast cancer. There have been only 5 reports so far [[1], [2], [3], [4], [5]]. In most cases, hemorrhage from the tumor results from tumor infiltration of the skin, and bleeding from the skin is rare when no tumor invasion is observed histopathologically [6,7]. Encapsulated papillary carcinoma (EPC) of the breast is an entity described in WHO classification of Tumours of the Breast (2012), and is considered to be non-invasive carcinoma or a low grade or indolent form of invasive carcinoma [8]. We report here a case of EPC with bleeding from the skin with no tumor invasion in which emergency surgery was performed to control bleeding, and verified the validity of emergency surgery. This work was written in accordance with the SCARE criteria [9].

## 2 Presentation of case

**Fig. 1:**
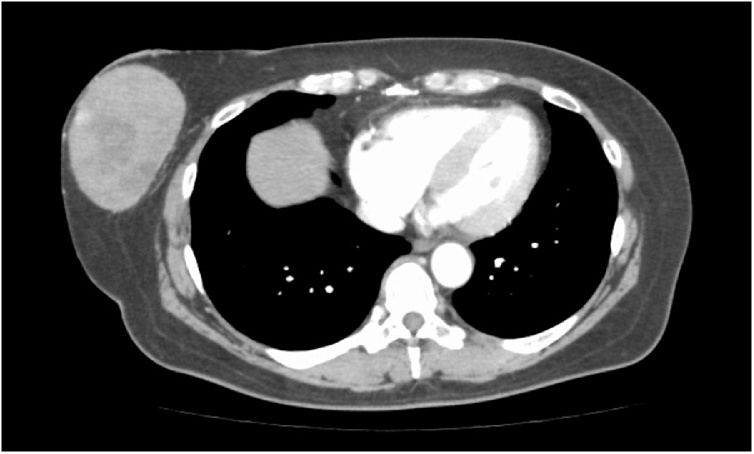
Contrast-enhanced CT showing a cystic mass of 9 cm in diameter in the right breast. The border is clear with accompanying thinning of the skin overlying the tumor.

**Fig. 2:**
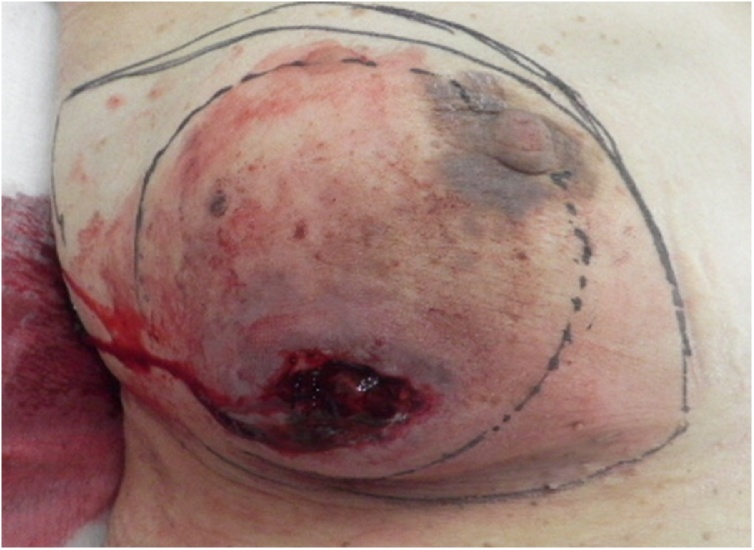
Palpable mass occupying the lower quadrants of the right breast. The skin overlying the tumor is breached, with accompanying bleeding.

**Fig. 3:**
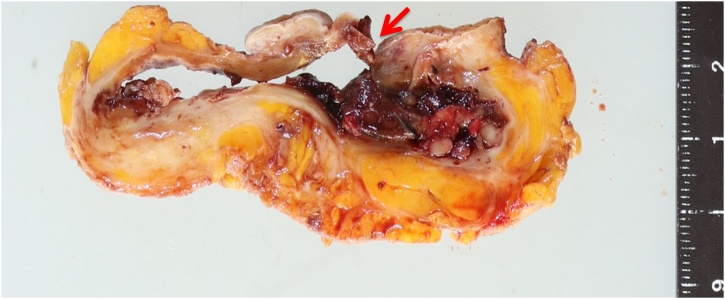
Gross appearance of a 7.5-cm cystic lesion with perforated skin. A solid mass (arrow) is observed in the lumen.

**Fig. 4:**
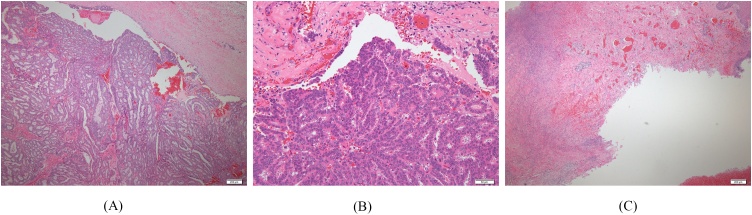
(A, B) Microscopy shows a tumor growing in a papillary pattern, with extensive bleeding. (C) The skin at the fistula is thin and discontinuous, but no surrounding tumor cells are seen.

A 62-year-old woman visited our hospital because of sudden bleeding from the right breast. One year earlier, she noticed a mass in the right breast, which gradually increased in size but had been left unattended. A few days before presentation, it increased rapidly in size and started bleeding. She was not on any anticoagulants or antiplatelet drugs. Physical examination revealed a tumor in the lower right breast measuring 9 cm in diameter. The skin directly overlying the tumor was partially thinned, necrotic, and bleeding. Further examination revealed a huge cavity inside the tumor, but no bleeding source was identified. Blood tests showed no significant abnormalities in blood count (hemoglobin 13.5 g/dL) and biochemistry; carcinoembryonic antigen was slightly elevated (9.7 ng/mL). Computed tomography (CT) revealed a clear cystic mass with a diameter of 9 cm on the right breast, with thinning of the overlying skin. Some areas of the tumors were hypervascular (Fig. 1). There was no pectoral muscle invasion or intraductal extension and no axillary lymphadenopathy. Mammography and ultrasonography could not be performed due to heavy bleeding. A hematoma was seen occupying the cyst, which was removed and the cavity was packed with gauze. Transient hemostasis was achieved but the bleeding resumed the next day. The skin defect was sutured closed and compression was reapplied with gauze, but hemostasis was not achieved. Blood count on hospitalization day 4 revealed anemia (hemoglobin 10.5 g/dL), and since the bleeding could not be readily controlled and further hemostasis was deemed difficult, emergency surgery was performed. Although embolization was cited as an option for treatment, resection was performed to ensure certainty. The aim of surgery was to achieve hemostasis, and no axillary lymph node search or dissection was performed; the tumor was excised with resection of the overlying skin (Fig. 2). Macroscopically, the excised tumor specimen was a 7.5-cm diameter cystic lesion with a solid mass in the lumen. Microscopically, atypical cells with enlarged nuclei were observed in the cysts growing in papillary and sieve-like patterns (Fig. 3). Growth of interstitial components was inconspicuous, and the tumor was considered to be of epithelial origin (Fig. 4A and B). There was no obvious infiltration of the surrounding stroma, but an accompanying ductal carcinoma *in situ* was seen in the surrounding mammary gland tissue. Surgical margins were negative. A fistula was seen with thin and discontinuous surrounding skin; no tumor cells were found (Fig. 4C) and it was deemed perforation due to retraction rather than an ulcer due to infiltration. The diagnosis was EPC according to the WHO classification. The subtype was Luminal A (estrogen receptor positive, progesterone receptor positive, human epidermal growth factor receptor 2 score 0, Ki-67 index 10 %). The postoperative course was good. After discharge, we instituted hormone therapy and the remaining breast tissue was subjected to radiation therapy. To date there has been no recurrence and no lymph node metastasis.

## 3 Discussion

Breast disease is rarely treated with emergency surgery. There are only 10 case reports of breast disease requiring emergency surgery in Japan [[1], [2], [3], [4]]. We found only 1 report on a case of emergency surgery performed due to bleeding from the breast in the international literature [5]. In most cases, emergency surgery was performed to control bleeding, and involved many phyllodes tumors and 4 cases of primary breast cancer. Only 2 cases of intracystic tumor, as in our case, have been reported [3].

In the differential diagnosis, various imaging diagnostics are performed, but the final result is often obtained from histological examination. If the whole lesion is a mass, imaging is useful to distinguish it from a phyllodes tumor. However, it is difficult to distinguish images with cystic lesions as in this case, and imaging tests such as magnetic resonance imaging (MRI) in the presence of persistent bleeding are not practicable.

EPC is an entity described in the WHO Classification of Tumours of the Breast (2012) and is considered to be non-invasive carcinoma or a low grade or indolent form of invasive carcinoma. Histologically, EPC is defined as papillary carcinoma with neoplastic epithelial cells exhibiting low core atypia with papillary growth pattern in a cavity surrounded by a macroscopic cystic fibrous capsule [8].

In our case, a sudden increase in the size of the mass was observed and effective hemostasis could not be achieved. This uncontrollable bleeding may have occurred due to a change in the tumor shape and a sudden rapid increase in the cyst size. The progressive bleeding could have led to skin crushing necrosis, resulting in massive hemorrhage.

We examined these processes pathologically and histologically, and our inferences were as follows:

1) The stromal vascular stem ruptured into the cyst and bled, with expansion of the free space in the cyst.
2) Enlargement of the free space increased the range of motion of the tumor, which further encouraged stromal vascular stem rupture and bleeding, leading to associated cyst growth.
3) With the enlargement of the cyst, skin necrosis occurred with fistula formation, and ensuing bleeding to the exterior.
4) There were likely multiple bleeding points within the cyst and hematoma, and so effective hemostasis could not be achieved *via* compression.

Puncture and drainage of the hematoma in the cyst is also considered an alternative to compression, but it has been reported that the cyst could return to its original size because the puncture would promote rebleeding [10].

For bleeding due to breast cancer, another option is bleeding control by endovascular embolization. Although some success has been reported [11], identifying the bleeding sources and achieving complete hemostasis is difficult; therefore, surgical intervention for hemostasis is often selected.

In this case, there was no vascular collapse on CT, anemia was absent, and hemorrhage was judged to be slow, so compression hemostasis was attempted repeatedly. However, when anemia was eventually noted, compression hemostasis was judged unlikely to be effective, and we opted for emergency surgery.

The purpose of emergency surgery for bleeding is to achieve hemostasis, but mastectomy and tumor resection must also be performed, meaning that surgery can also treat the tumor.

For patients, it is natural that an optimal surgery without excess or deficiency is desired, and as much preoperative diagnosis as possible is vital.

Diagnosis of breast cancer in daily practice is based on mammography and ultrasonography, and metastasis is detected using contrast-enhanced MRI and positron emission tomography. However, mammography is not feasible with persistent bleeding and a large mass, and even with ultrasonography it would be difficult to visualize the details of an already enlarged tumor in such cases. Therefore, diagnosis with contrast-enhanced CT is the main option.

From past case reports, sudden bleeding from the breast is assumed to be mainly phyllodes tumor and breast cancer. Bleeding is presumed to be from rupture due to tumor progression [11] and rupture of blood vessels into cysts [10]. In our case, the rapid tumor growth and bleeding suggest vascular breakdown into the cyst.

To our knowledge, there are no reports on prognosis in breast cancer patients following emergency surgery due to hemorrhage, and prognosis was therefore determined based on the final pathological diagnosis, with consideration of the postoperative course. In this case the diagnosis was EPC, which usually has a good prognosis.

In routine clinical practice, sentinel lymph node biopsy is performed except in cases with obvious metastasis, but this is practically impossible in emergency surgery, and thus was not done in this case. On the other hand, when there is bleeding from the lesion in the lymph node region, axillary lymphadenopathy if present may be due to reactive enlargement. Reassessment of the axillary lymph nodes after surgery may be essential.

For distant metastases, preoperative evaluation in cases requiring emergency surgery could be inadequate. In our case, CT was repeated after surgery with careful follow-up using ultrasonography, but no findings suggestive of lymph node metastasis or distant metastasis were seen.

Furthermore, there could be tumor cells in the shed blood, and this carries a high risk of contamination during surgery. Therefore, more thorough observation for local recurrence than usual is necessary. If exposure of surrounding tissue to tumor cells is expected, additional radiation therapy may be considered even for cases of total mastectomy.

## 4 Conclusion

We have reported a case of EPC that required emergency surgery due to hemorrhage that could not be controlled by compression. Emergency surgery is rare in the management of breast disease, but should always be considered as an option when bleeding cannot be readily controlled. Preoperative evaluation and further postoperative evaluation are vital in performing emergency surgery, particularly in terms of postoperative treatment.

## Funding

Our study has not received any grant of funding.

## Ethical approval

Our institution does not require ethical approval for a case report that are deidentified and collected retrospectively.

## Consent

Written informed consent was obtained from the patient for publication of this case report and accompanying images.

## Author contribution

Kimiyau Yoneyama contributed to operation and writing the manuscript.

Asuka Hara contributed to operation.

Motohito Nakagawa reviewed the work.

## Registration of research studies

This is a case report, and no database approval was applied.

## Guarantor

Kimiyasu Yoneyama.

## Provenance and peer review

Not commissioned, externally peer-reviewed.

## Declaration of Competing Interest

The authors declare that there is no conflict of interest regarding the publication of this article.
